# Effect of having and switching multiple avatars on the operator’s right to talk and receive social support

**DOI:** 10.1371/journal.pone.0292803

**Published:** 2023-10-16

**Authors:** Faisal Mehmood, Hamed Mahzoon, Yuichiro Yoshikawa, Hiroshi Ishiguro

**Affiliations:** 1 Department of Systems Innovation, Intelligent Robotics Lab, Graduate School of Engineering Science, Osaka University, Suita, Osaka, Japan; 2 Institute for Open and Transdisciplinary Research Initiatives (OTRI), Osaka University, Yamadaoka, Suita, Osaka, Japan; COMSATS University Islamabad, PAKISTAN

## Abstract

People with communication difficulties encounter several challenges in their daily online interactions, such as a limited right to talk (RoT), insufficient social support (SS), and a low sense of being attended to (SoBA). Computer-mediated technologies are limited in addressing such problems owing to their limited capacity in transferring verbal and nonverbal cues between users. In this study, to address the limited RoT, low SS, and low SoBA challenges, we proposed a robotic video conference system with two teleoperated robot avatars. The proposed system was compared with another robotic video conference system that adopts only one teleoperated robot avatar. In the field experiment, 37 participants took part in two discussion sessions using each system type, where RoT, SS, and SoBA were adopted as the measured indices. The proposed system significantly increased the users’ RoT and SS compared with other robotic video conference systems. This study contributes to the literature by demonstrating the effect exerted by the type of robotic video conference adopted on users’ feelings about RoT, SS, and SoBA.

## 1 Introduction

Individuals with communication difficulties include the ones who are apprehensive about communication and encounter several problems in their daily online interactions [1–3]; such as limited right to talk (RoT), lack or absence of social support (SS), and low social presence (SP) in communication. The first stated problem, RoT, is defined as an individual’s feelings concerning the provision of equitable speaking and opinion-expressing opportunities relative to their peers in conversation. Such feelings are influenced by different types of elements in their conversations, such as the number of conversational turns, duration of listening and talking [4], number of utterances, inter-utterance pauses, and back-channel responses [5]. Ideally, equality is required among all types of elements for each conversation peer as general rules of communication. However, in reality, such rules are severely prone to violations that trigger vocal interruptions, speech disruptions, and social anxiety among peers [6]. The risk of violating these rules can be significantly minimized if conversation peers socially support each other. The second aforementioned problem, lack or absence of SS, is defined as information that leads the subject to believe that he is cared for, loved, esteemed, and considered a member of a network with mutual obligations [7]. In conversations, the presence of SS increases the willingness to communicate with individuals [8] and minimizes anxiety and depression [9]. Finally, the third aforementioned problem, low SP, is defined as an individual’s perception of their presence in a conversation [10]. It contributes to the feelings of belonging and connectedness in a conversation [11,12] Further, such feelings influence an individual’s sense of being attended to (SoBA), which is defined as the experience of receiving appropriate focus and attention in a conversation, including being listened to and answered [13].

Computer-mediated communication (CMC) technologies have been investigated to resolve the challenges of limited RoT, lack or absence of SS, and low SP issues for individuals with communication difficulties. CMC includes text, audio, and video interaction technologies. Although each CMC technology provides a huge support to enable people with communication difficulties establish effective communication with others, these technologies fail to assure the RoT of users owing to their limited ability to transfer verbal and non-verbal information that influences the adherence to communication rules [14]; hence, users need to reconcile RoT via mutual cooperation [15,16]. Furthermore, CMC technologies are limited in offering SS to users [17]; for example, text-only technology is significantly limited in offering SS [18]. However, audio-only technology can provide limited SS [19], because in addition to verbal cues, it also provides limited non-verbal cues, such as vocal tone variation associated with discrete emotions [20]. In contrast, video technology is relatively better at providing SS to users [21] because, in addition to verbal cues, it also provides limited non-verbal cues such as awareness of attentional focus, ease in speaking turns, and facial expressions [22,23]. Apart from SS, CMC technologies are limited in providing a SP to users [24]. Text-only technology provides a severely low SP to users [24,25], while audio-only technology provides a low, but better SP than text-only technology [26]. Although video technology is relatively robust in providing SP to users [27,28], the presence of video streaming causes an increase in the communication apprehension of users, which eventually decreases the ease of talking [29–31]. To prevent such problems while maintaining SP, we must explore other available technologies. Considering the performance of text- and audio-only technologies regarding the provision of SP to users, they are also expected to be severely limited in providing SoBA to users. However, video technology can provide a significant amount of SoBA to users, as it provides a rich social presence [32]. In conclusion, text, audio, and video technologies are all limited in their ability to address the aforementioned problems.

Robot avatar technology has been investigated considering SP; however, the RoT and SS challenges in communication are yet to be studied. There are two types of robot-avatar technology users: those interacting through avatars (hereafter referred to as operators) and those interacting with avatars (hereafter referred to as visitors). In social interactions, using a single physical avatar facilitates communication between operators and visitors, such as an interaction between a teacher and a student in an educational environment [33,34] and an interaction between two family members [35,36]. However, such interactions are dyadic in nature; because on the visitor’s side, only a single physical avatar and a visitor interact with each other, while on the operator’s side only an operator and a visitor interact with each other. In other words, the ratio of amount of talk of each person needs to be 1/2 in dyadic interaction i.e., equal number of utterances, equal number of words, and equal inter-utterance pauses [37]. However, based on the personality of the person, such an aspect varies from person to person, so it is difficult to increase the operator’s RoT; especially when he/she has communication apprehension. Therefore, this raises the following research questions: In an avatar-mediated communication, is there any way to manipulate feelings of RoT in operators? Such a question is yet to be explored. Experiences via avatars are considered the operators’ experiences [38], and when communicating with a visitor via an avatar, the operator cannot avail SS from their own robot avatar. Because a dyadic interaction exists with the visitor via the avatar, the operator can only avail limited SS. The feelings of receiving SS are crucial in interactions, especially for people with communication difficulties [39]; therefore, alternative methods are required. A human subject can experience a high SoBA by watching a video scene of a conversation, where a visitor interacts with the avatar of a side-participant [13]. This implies that an operator feels supported in conversation via the avatar when another avatar cares for their avatar. The usage of two avatars is useful from visitors’ point of view e.g., 1) producing coherence in conversation [40], 2) improving social behaviors [41], 3) generating pressure in communication [42] and, 4) persuading for certain actions [43]. Now, it is important to investigate the effect of adopting a second avatar on the operator communicating via an avatar. The visitor talking with the two avatars of the operator would be engaged in a triadic interaction; where, as per triadic interaction rules of talk, the ratio of amount of talk for each one needs to be 1/3 of total amount of talk [37]. As both robots would be the avatars of an operator so, it is expected that operator’s RoT will increase according to the rule of communication for triadic interaction. Moreover, there is a possibility of including SS for an operator from avatars in such triadic interaction. Therefore, in this study, we propose a novel system comprising two avatars and controlled by an operator, to communicate with the visitor and experience higher RoT and SS.

**Fig 1:**
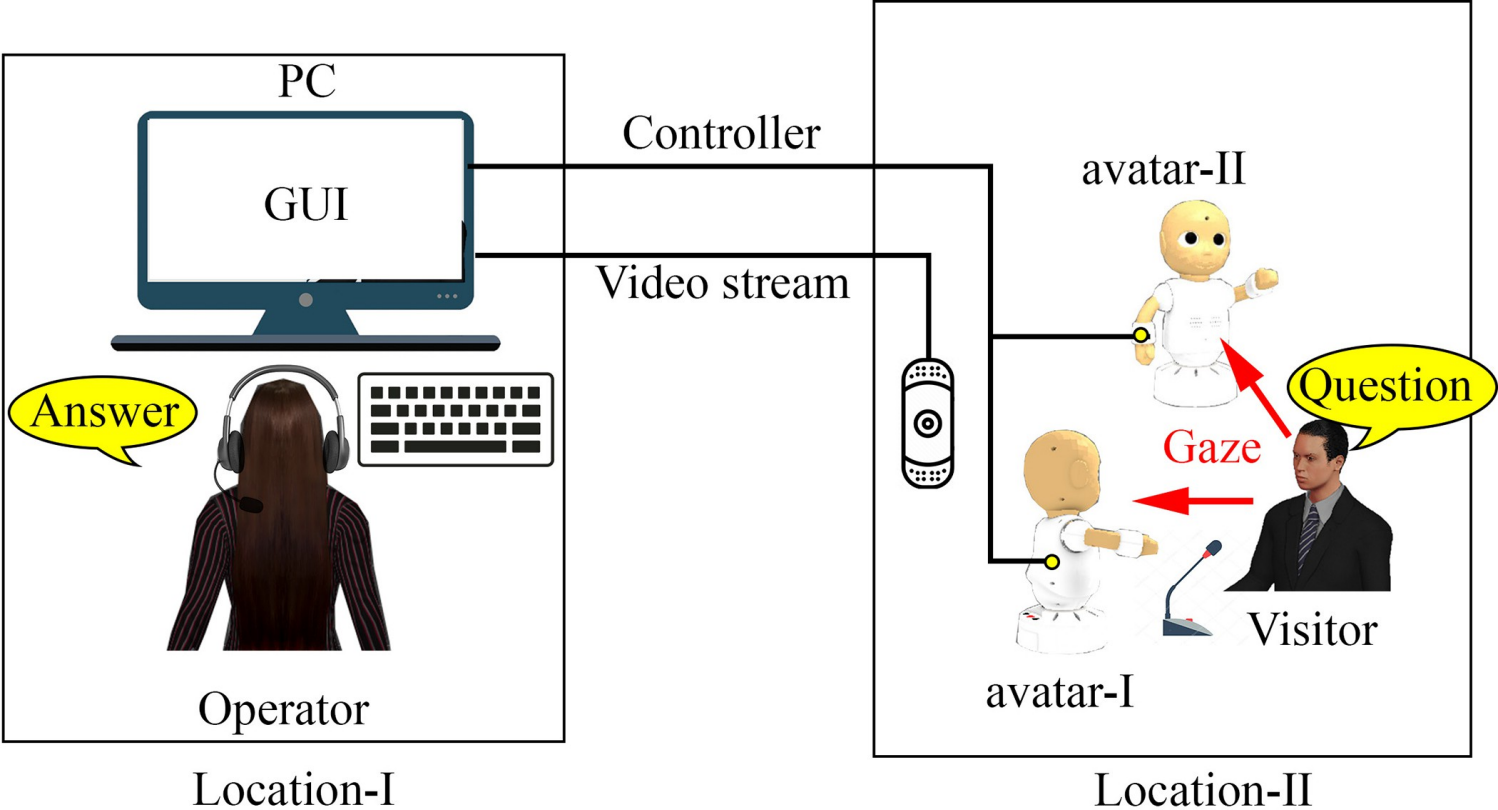
Proposed robotic system with two teleoperated robot avatars.

In the proposed system (refer to Fig 1), the operator’s utterances emerge from either of the teleoperated avatars. The choice of the speaking avatar and the production of backchannel responses from the other avatar were processed randomly. The frequency of switching avatars was depending on how often an operator wants to utter; where switching of avatars was triggered by typing or speaking of the operator. Similarly, the timings of switching of avatars was based on the operator’s speed of preparing utterances. Sometimes, the second avatar took a speaking turn from the current avatar to talk about the same opinion as the first avatar; as the speaking content is defined by operator only. Such turn-taking and backchanneling behaviors of the second avatar are expected to make the operator feel supported in communication. Consequently, the visitor also had to switch attention towards the speaking robot avatar throughout the conversation. Such approach by the visitor was evident to the operator by the video feed on the monitor. Furthermore, the proposed system is expected to provide another advantage of increased RoT because an operator is expected to attend a multiparty conversation with two agents. Humans usually tend to equalize conversational turns, time to listen to and talk [4], including the number of utterances, inter-utterance pauses, and back channel responses in conversation [5]. This indicates that humans tend to expect an equal RoT for each participant during the conversation. Because experiences via the avatars are considered the operator’s own experiences [38], the assigned RoT of each avatar is expected to be perceived by the operator as their own RoT in a multiparty conversation. Therefore, in the proposed system, the operator is expected to perceive a maximum of double RoT levels. Conversely, at least more than one is expected to be perceived when communicating via a single avatar.

## 2 Video teleconferencing system involving physical avatars

A schematic of the proposed system is presented in Fig 1. It comprises a computer, a monitor screen, a headset with a microphone, a keyboard, two semi-humanoid robots, and a web camera. The components used to develop the proposed system were ordinary that were commercially available easily. Using a computer and web camera, an online interaction session was arranged between the operator and visitor physically present at different locations (Locations I and II). The robots were physically present at Location II, one in front and another one beside the visitor. Both robots were avatars of the operator. We utilized the CommU robot, which was developed via collaboration between Osaka University and Vstone Co., Ltd., Japan [44]. The CommU robot is a semi-humanoid robot with clear eyes of 14 degrees of freedom in total and a 31-cm height; in addition, it is programmable using the JavaScript language and can interact via visual, speech, and motion stimuli.

**Fig 2:**
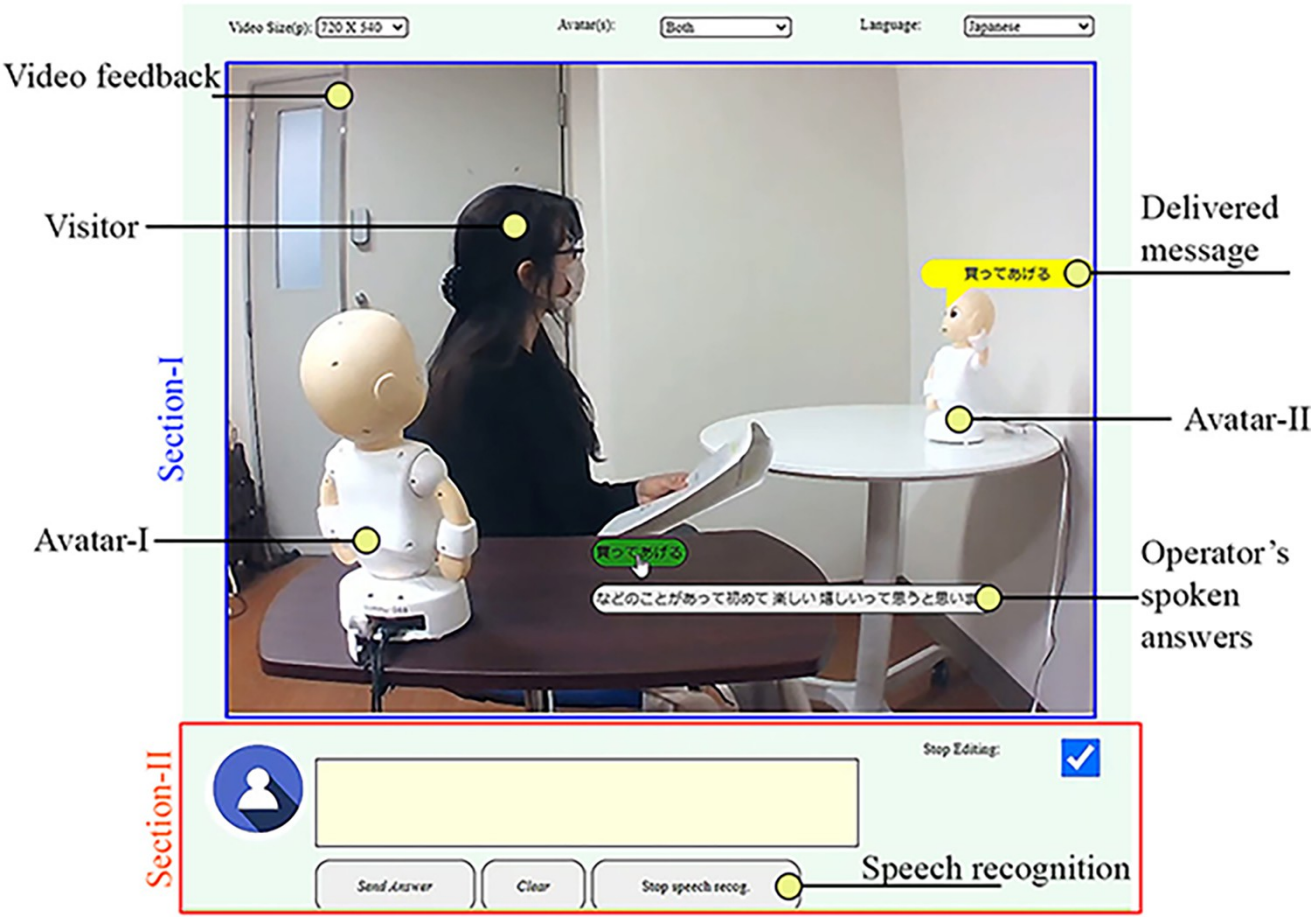
GUI on the operator’s computer screen.

To control the robot avatars over a wide area network in real time, a locally built graphical user interface (GUI) was adopted by the operators, as illustrated in Fig 2. The GUI comprises two sections: Sections I and II with visual feedback and utterance-related handling-options, respectively. The visual feedback section was designed using web-RTC, which provides a real-time view of the visitor’s environment to the operator, referred to as Section I in Fig 2. It also displays the detected spoken answers of the operator in the middle of the section as dynamically added buttons at the bottom; where to detect and to convert the spoken answers into text, a Web speech API, written in java script programming language, was used. However, the utterance section comprises a text field with three buttons, referred to as Section II in Fig 2. It provides several facilities to the operator, such as typing new answers, editing previously detected answers, deleting answers, and enabling/disabling speech recognition.

In an online interaction session, the proposed system behaves in two different ways in which an operator provides the answer(s) to the visitor’s question(s). In a case where the operator does not provide the answer(s), Avatar-I remains still with a visual focus toward the visitor, while Avatar-II continues to perform idling motions, i.e., the avatar keeps switching visual focus between the visitor and Avatar-I by turning its head and torso. However, in a case where the operator provides the answer(s), one of the avatars utters in a synthesized voice in front of the visitor while raising their left arm. Meanwhile, the other avatar shifts its visual focus to the uttering avatar and acknowledges them by nodding, thus pretending that the provided answer is accurate and acceptable. Then, the system randomly chooses an avatar to deliver the operator’s answer. While conversing via such a system, the visitor asks questions from the avatar who delivers the answer and the operator is required to answer the questions either by speaking or typing. The proposed system also manages conversational turns between the operator and visitor. When the visitor speaks, the operator obtains information from a real-time video feedback. However, when the operator types or speaks the answer, the visitor obtains information from the glowing (red) cheeks of the robot(s).

## 3 Materials and methods

### 3.1 Method

The impression of the conversation between the operator and visitor was evaluated using two types of systems: the conventional and proposed systems. The conventional system is a video conference system integrated with a single teleoperated avatar (hereafter referred to as the single-avatar condition), whereas the proposed system is a video conference system integrated with double teleoperated avatars (hereafter referred to as the double-avatar condition). The recruited participants were asked to physically visit the experimental site and attend four online conversation sessions with a visitor i.e., two practice sessions and two experimental sessions. Both practice and experimental sessions had two conditions: single- and double-avatar conditions. The purpose of inclusion of practice sessions was to familiarize the participants with operations of conventional and proposed systems and even provide them a chance to ask questions related to operations of both systems; if there is an ambiguity in operating both systems real-time. The number of avatars was an independent variable in this study, while the RoT, SS, and SoBA were dependent variables. At the end of the experiment, we interviewed the participants to know about the weakness and strengths of double avatar condition.

### 3.2 Participants

We recruited thirty-seven native Japanese-speaking participants (M = 21.68 years, SD = 2.13 years), which included twenty-one males and sixteen females. The participants were randomly divided into two groups: G1 and G2. Group G1 experienced the single-avatar condition first, followed by the double-avatar condition; however, the experiencing sequence was the opposite for group G2. It should be noted that the practice sessions are followed by experimental sessions. Practice sessions consist of two counterbalanced conditions i.e., single avatar and double avatar. Similarly, experimental sessions also consist of two counterbalanced conditions i.e., single and double avatar.

### 3.3 Conversational scripts

#### 3.3.1 Practice sessions

We chose two short conversational scripts for the practice sessions and asked the participants for their recommendations. In the first short conversation script, recommendations were related to the type of food, whereas in the second short conversation script, recommendations were related to club activities in a schooling period.

#### 3.3.2 Experimental sessions

To choose conversational scripts for the experimental sessions, we conducted a separate subjective evaluation experiment in which the forty-seven recruited participants (M = 40.27 years; SD = 8.12 years) read and evaluated four different conversational scripts regarding RoT and SS. Subsequently, we chose two conversational scripts with equal RoT and SS values. The topics of the chosen conversational scripts were 1) “should a person choose love or money to live a better life?” and 2) “should a person save the life of a child or the lives of two old persons in a car accident?”. The criteria of selection of scripts were as follows: 1) the script should not have a controversial topic, 2) the scripts should have equal length and equal number of branches, 3) the script should include a topic with tricky choices for answer(s), and apparently there should be no right or wrong answer, and 4) the scripts should include balanced arguments and counter-arguments so that the chances of having feelings of winning over or losing against an opponent is minimized.

### 3.4 Stimuli

In both experimental sessions, an operator (i.e., participant) and a visitor talked about two topics: “whether a person should choose love or money to live a better life?” and “whether a person should save the life of a child or the lives of two old persons in a car accident?” Similar to participants, we separately hired three female actors who played the visitor’s role in our experiments; aged in-between forty-five to fifty-five years. The contents of these topics are presented in the supporting information. Please note that conversational topics were counterbalanced between experimental sessions. In a single avatar-based conversation session, an avatar agent (Avatar-I; Fig 2) was placed in front of the visitor and teleoperated by the operator to convey answers to the visitor. During the conversation, the visitor directed their attentional focus to the avatar agent, while both the visitor and teleoperated avatar agent were visible to the operator through the monitor of the video conference system. In the double avatar-based conversation session, two teleoperated avatar agents were placed: one on the left side and the other in front of the visitor, as illustrated in Fig 2. The operator’s answers were produced randomly by either of the robot avatars and conveyed to the visitors in a synthesized voice. During the conversation, the visitors kept changing their attentional focus by turning their head and torso toward the speaking avatar agent. Meanwhile, both the visitor and teleoperated avatar agents were visible to the operator through the video conference system monitor. In both experimental sessions, in addition to asking questions from the operator, the visitor also provided logical reasoning, such that the operator thought about changing their opinion. The sequence of questions and the provision of logical reasoning remained the same in both conditions. The duration of each practice session was approximately 2–3 min. However, for each of the experimental sessions, it was approximately 10–12 min. The language of the conversation practice and experimental sessions was Japanese.

### 3.5 Procedure

The participants were required to visit the experimental site where they read and agreed to the content of the written consent form. Meanwhile, they were randomly assigned to either G1 or G2. In the beginning, participants were required to complete two short practice sessions, where they practiced the usage of both systems, i.e., the single- and double-avatar conditions. They were briefed on the functionalities of each element of the GUI controller and later filled out the questionnaire forms. After completing the practice sessions, the participants were required to complete two experimental sessions. In the experimental session, the participants in Group G1 were briefed again regarding the single-avatar condition. In the briefing, the functionalities of all elements of the GUI and the topic of conversation were explained to them. They were also instructed not to rush and then provided with very long answers. They were advised to use the system peacefully, reply calmly, and as attempt to provide as many short answers as they want. Subsequently, the first experimental session was arranged, where the participants experienced the conversation using a single-robot condition and filled out the questionnaire form. Similarly, a second experimental session was arranged in which the participants experienced the conversation via the double-robot conditions and later filled out another questionnaire form. However, for the participants in G2, the sequence of experience of conditions was the opposite.

### 3.6 Measurements

#### 3.6.1 RoT

RoT refers to an individual’s feelings concerning the provision of equitable speaking and opinion-expressing opportunities relative to those of their peers in a conversation. The concept of RoT can be drawn from the previous literature [45,46] which provides the information about the personality characteristics of individuals with communication apprehension in face-to-face communication. However, such a concept is not explicitly mentioned. So, to the best of our knowledge, the concept of RoT is relatively new in the context of communicating through teleoperated avatar(s). We developed a novel scale in Japanese to quantify such feelings in the conversational scenario of our experimental setup. To appropriately quantify an individual’s feelings of equitable speaking and opinion-expressing opportunities, we are required to provide him/her a chance of high self-reflection; possible, if an appropriate number of response categories are used in the scale, e.g., seven categories [47]. So, a 1–7 Likert-type point scale was adopted (strongly disagree, somewhat disagree, disagree, neither agree nor disagree, somewhat agree, agree, strongly agree), where ratings were summed to yield the operator’s total scores for both conditions. The corresponding English translation of the questionnaire is provided into the supporting information.

#### 3.6.2 Validity and reliability of RoT scale

The validity and reliability of the RoT scale were assessed via a separate subjective evaluation experiment in which fifty-two recruited participants (M = 42.67 years; SD = 9.09 years) watched two video stimuli and rate their expected feelings of RoT. After obtaining data from subjective evaluations, an exploratory factor analysis was conducted. The Kaiser-Meyer-Olkin (KMO) test indicated its sample adequacy (KMO = 0.91) and Bartlett’s tests of sphericity revealed the factorability of the covariance matrix (*X*^*2*^(15) = 966.47; *p* < 0.05). Principal component analysis was utilized for factor extraction. Following the Kaiser criterion of factor(s) retention, only one factor was retained, indicating 87.61% of the total variance. All items on the RoT scale were significantly loaded for the retained factor. The reliability of the RoT scale was measured using Cronbach’s alpha, which was determined to be significantly high (α = 0.97).

#### 3.6.3 SS

SS is an information that leads the subject to believe that he is cared for, loved, esteemed, and considered a member of a network with mutual obligations [7]. We developed another novel scale in Japanese to quantify such feelings in the conversational scenario of our experimental setup. Since the concept of SS is known in the literature, so we need to focus on reducing confusion and increasing response rate for participants; hence a 1–5 Likert-type point scale was adopted (strongly disagree, disagree, neither agree nor disagree, agree, and strongly agree) [48]. The ratings were summed to yield the operator’s total scores for both conditions. The corresponding English translation of the questionnaire is provided into the supporting information.

#### 3.6.4 Validity and reliability of SS scale

The validity and reliability of the SS were assessed by conducting a separate subjective evaluation experiment in which fifty-two recruited participants (*M* = 42.67 years; *SD* = 9.09 years) watched two video stimuli and rate their expected feelings of SS. After obtaining data from subjective evaluations, an exploratory factor analysis was conducted. The Kaiser-Meyer-Olkin (KMO) test indicated sample adequacy (KMO = 0.85) and Bartlett’s tests of sphericity revealed the factorability of the covariance matrix (*X*^*2*^(6) = 582.63; *p* < 0.05). Principal component analysis was adopted for factor extraction. Following the Kaiser criterion of factor(s) retention, only one factor was retained, exhibiting 91.77% of the total variance. All items on the SS scale were significantly loaded for the retained factor. The reliability of the SS scale was determined using Cronbach’s alpha, which was considered significantly high (α = 0.96).

#### 3.6.5 SoBA

SoBA is a scale used to quantify the feelings of a participant concerning being listened to, attended to, focused on, or questioned/answered by an individual in a conversational scenario; the SoBA adopted here was developed by [13]. We updated the SoBA questionnaire according to our experimental setup while retaining the essence of the original scale and translated it into Japanese. Later, we calculated the reliability of SoBA for both conditions i.e., single avatar (α1 = 0.825), and double avatar (α2 = 0.863); obtained average reliability; i.e., α_average_ = (α1+α2)/2; α_average_ = 0.84. A 1–5 Likert-type point scale was adopted (strongly disagree, disagree, neither agree nor disagree, agree, and strongly agree), where the ratings were summed to yield the operator’s total scores for both conditions. The corresponding English translation of the questionnaire is provided into the supporting information.

## 4 Results

### 4.1 Software

We used IBM SPSS Statistics 26 software to perform all of the statistical analyses reported in the manuscript.

### 4.2 Normality test for data

The type of distribution of data for RoT, perceived SS, and SoBA was assessed by Shapiro-Wilk tests. The results of the tests showed that type of distribution of data for RoT and perceived SS was not normal while for SoBA, it was normal. Hence, we chose non-parametric statistics for RoT and perceived SS while parametric statistics for SoBA.

### 4.3 RoT

**Fig 3:**
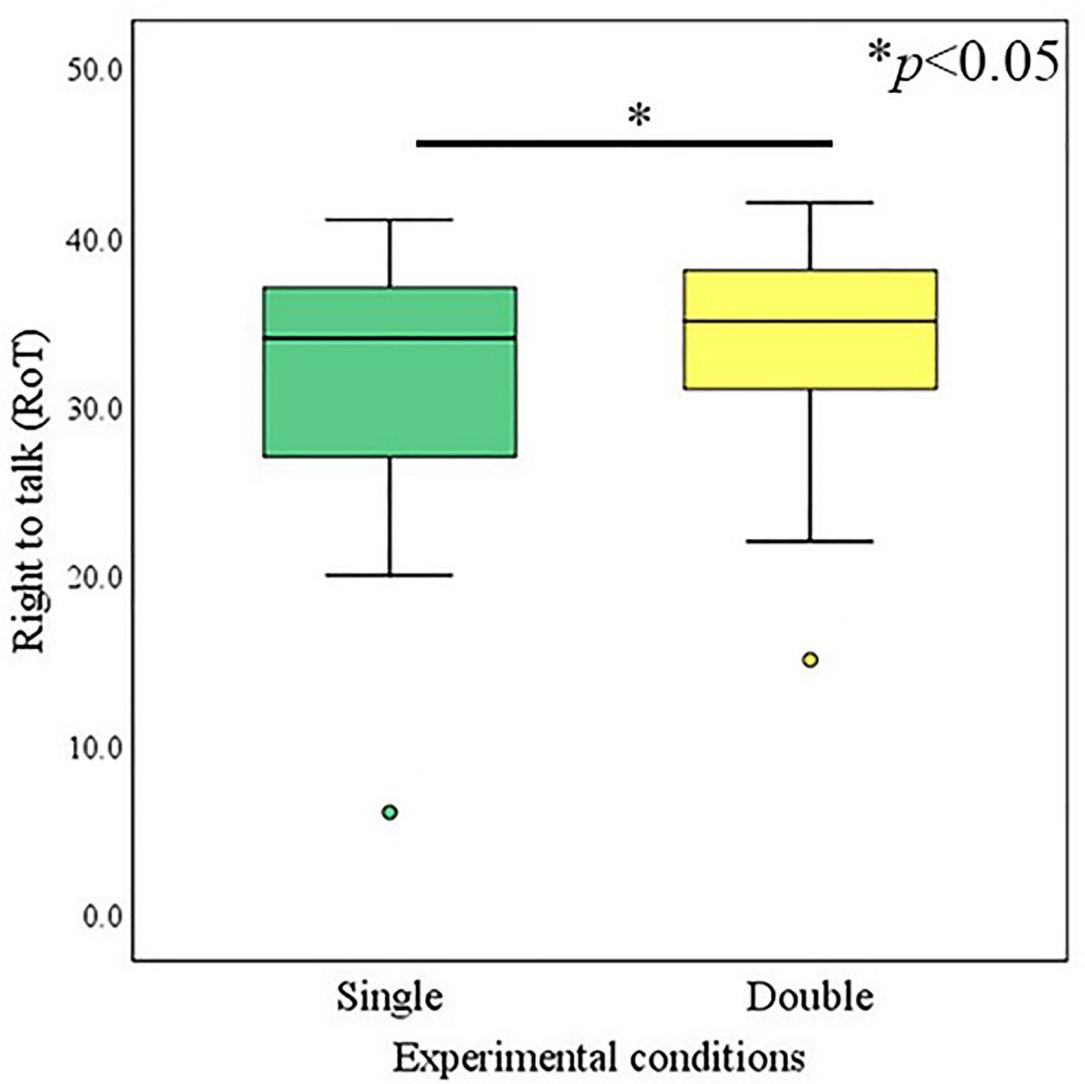
RoT of operators.

The Wilcoxon-signed rank test was conducted to identify the effect of the condition adopted (single avatar vs. double avatar) on the operator’s RoT feelings. The median value of the RoT for the operator of the double-avatar condition (*Mdn* = 35) was significantly higher than that for the single avatar condition (*Mdn* = 34) (*Z* = -1.99, *p* = 0.047, *r* = 0.23), as presented in Fig 3.

### 4.4 Perceived SS

**Fig 4:**
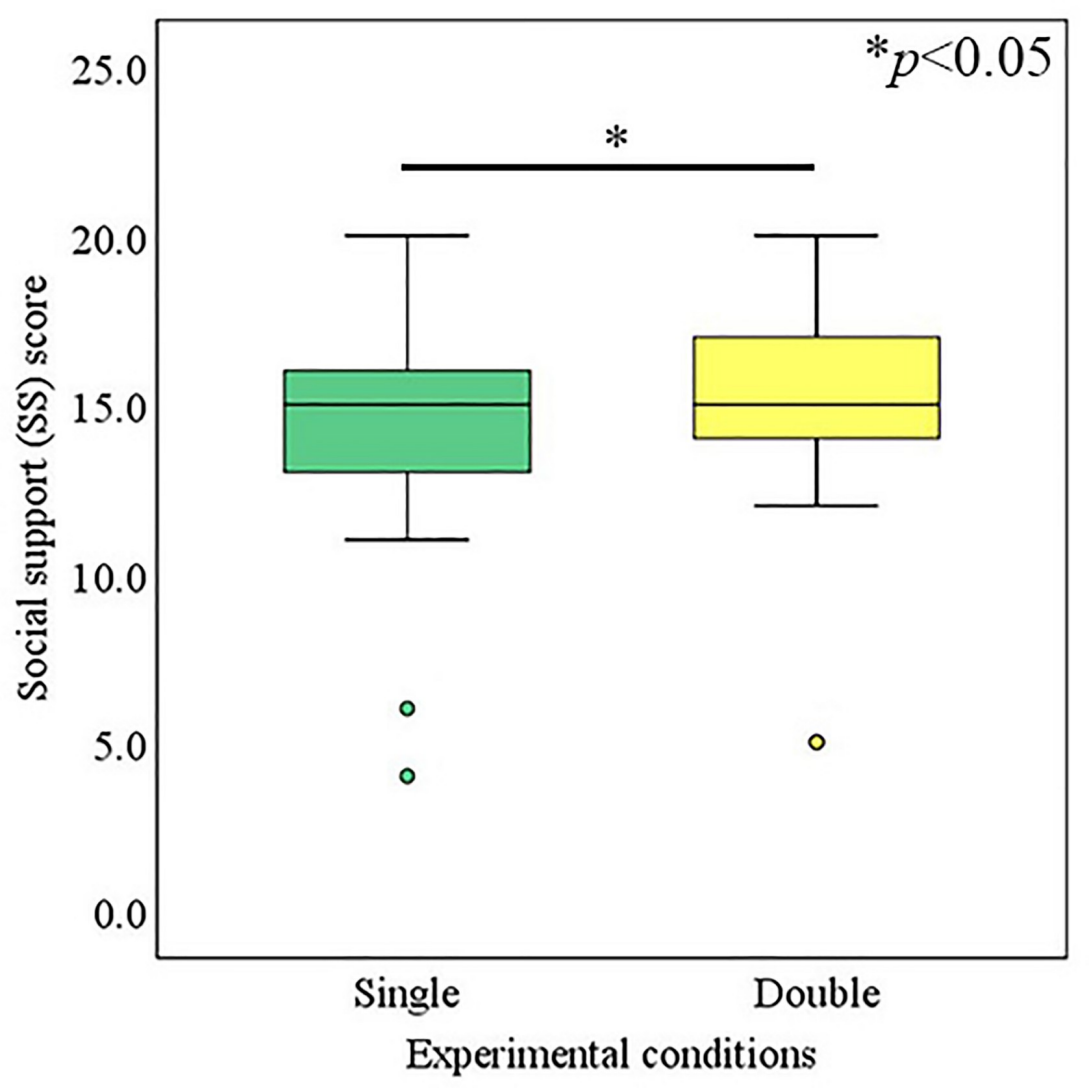
SS of operators.

The Wilcoxon-signed rank test was conducted to identify the effect of the condition adopted (single avatar vs. double avatar) on the SS of the operator. It was demonstrated that the median value of the SS for the operator of the double-avatar condition (*Mdn* = 15) was significantly higher than that for the single avatar condition (*Mdn* = 15); (Z = -2.11, *p* = 0.034, *r* = 0.24), as presented in Fig 4.

### 4.5 SoBA

**Fig 5:**
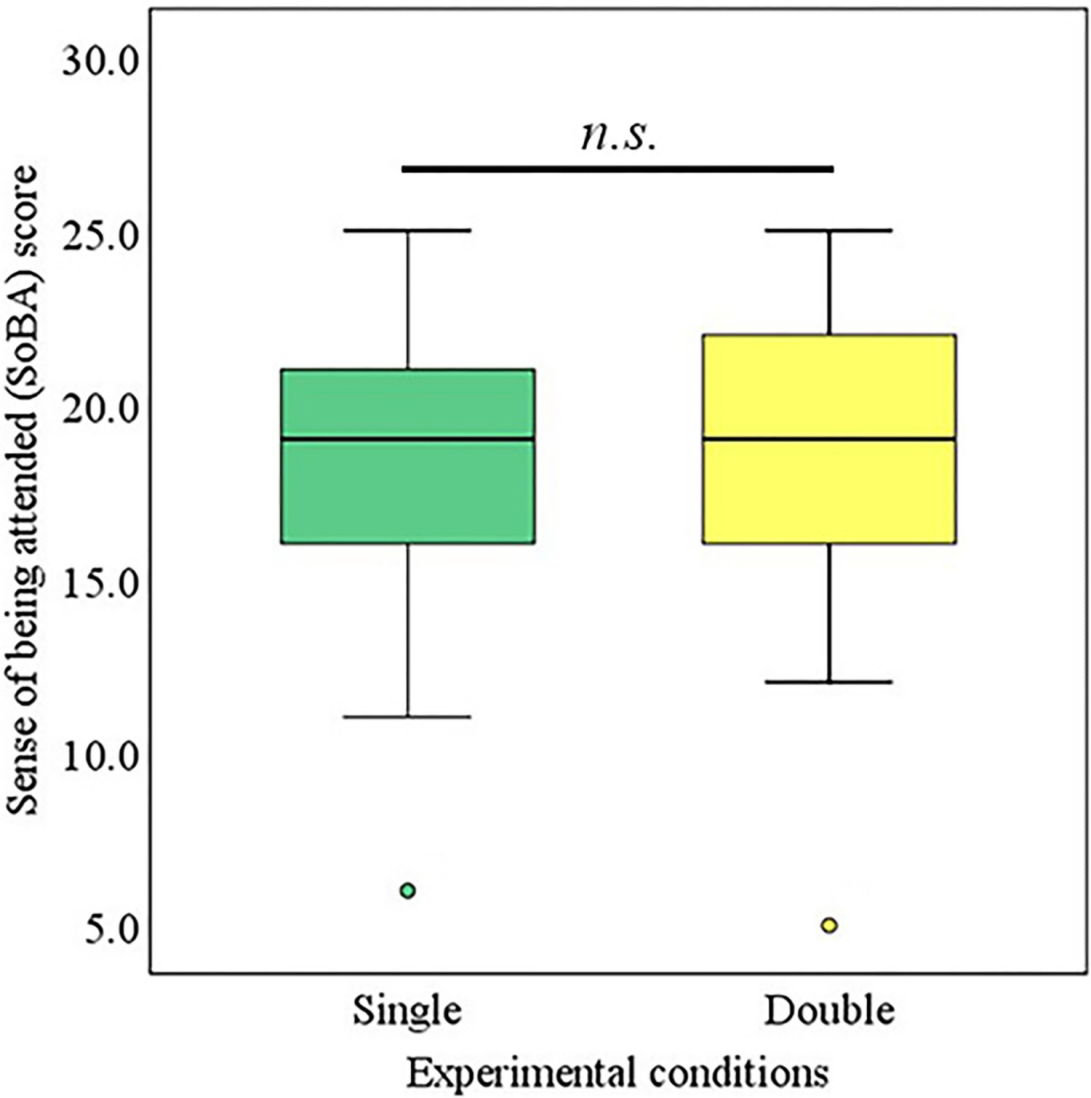
SoBA of operators.

Two paired-sample t-tests were conducted to identify the effect of the type of condition adopted (single avatar vs. double avatar) on the SoBA of the operator. The mean value of the experienced SoBA for the double-avatar condition (*M* = 18.29, *SD =* 4.26) was not significantly higher than that for the single avatar condition (*M* = 18.13, *SD =* 4.06), (*t (36)* = -0.33, *p* = 0.74, *d* = 0.054), as illustrated in Fig 5.

## 5 Discussion

Experimental results demonstrated that operators talking via double avatars experienced relatively higher communication support compared to those talking through a single avatar, and communication support was assessed by RoT, perceived SS, and SoBA indices. Operators with double-robot avatars perceived significantly higher SS in communication compared to operators with a single-robot avatar. Similarly, operators with double-robot avatars felt significantly more RoT in conversation compared to operators with a single-robot avatar. However, there was no significant difference between the SoBA of operators using single- or double-robot avatars. It is inferred that the use of double avatars provides communication support to operators by manipulating their feelings of RoT and increasing their perception of SS in conversations.

The primary reason for the significant increase in RoT was the operator’s feeling of owing the individual RoTs of their two remote representations i.e., their avatar agents. Humans tend to follow the rules of conversation [4,5]. In our conversation setup in which a visitor interacts with two avatars simultaneously, the conversation is perceived as a triadic interaction scenario by the operators. In such a triadic interaction scenario, an equal RoT is received by each peer of the conversation, i.e., the visitor and avatars. However, because the operators teleoperated the avatars, and experiences via avatars are considered their own experiences [38], the individual RoT of each avatar would eventually become the operator’s RoT; the main cause of the observed effect significantly increased the RoT feelings of the operators. Such an accumulative RoT would have a maximum of up to two-fold. An important point to keep in mind for obtaining the less effect of RoT for operators than the maximum i.e., two-fold could be explained by the varying degree to which an operator accepts the individual RoT of each avatar as his/her own RoT. The primary reason for the significant increase in SS was the operator’s feeling of being supported by avatar agents in front of the visitor throughout the conversation, even when there was a difference in opinions. SS refers to the information transferred to a subject that leads them to believe that they are cared for, loved, esteemed, and considered a member of a network with mutual obligations [7]. How and in which specific time frames such information is being transferred are crucial elements influencing the beliefs of subjects [49]. In general, such information can be transferred to the subject by exhibiting verbal, non-verbal, or both reactions, i.e., verbal and non-verbal behaviors in a specific time frame. Here, the robot avatars transferred such information by exhibiting non-verbal supportive behaviors to the operators in front of the visitors, specifically at the time when they finished expressing their opinions. Such supportive behaviors were displayed to the operators throughout the conversation session, which was the major cause of the observed effect, i.e., the significantly high perceived SS.

Operators talking via double avatars were expected to have a significantly higher SoBA compared to operators talking through a single avatar. However, this effect was not observed in this study. Instead, a negligible increase in the SoBA was observed. Two possible reasons can explain the absence of such an effect on the operators: 1) differences in the treatments of the visitors, and 2) the effect of partial occlusion in the field of view for the operator. In remote interactions, SoBA is more related to the treatment of visitors towards the operator’s remote representations i.e., avatar robot(s). Such treatments were expected to be perceived by operators as treatment for themselves. However, as the visitors differ from each other in terms of reproducing similar behaviors as instructed, so it might be possible that the operators did not receive similar types of treatments. Therefore, it is quite natural to accept such a fact because the degree to which the given instructions are strictly followed varies among visitors i.e., humans [50–52]. Moreover, one of the avatars was placed in the line of sight of the operator i.e., in front of the camera in such a way that the rear side of the head and torso was evident, thereby triggering a small degree of visual occlusion in the field of view of the operator. For operators, such a visual occlusion hinders the process of direct visual attention from visitors and eventually influences the quality of interaction [53]. Therefore, the presence or absence of direct visual attention from visitors influences the SoBA of operators [13].

Despite communication support via double physical avatars, a few limitations remain. First, the degree to which each participant perceives a lack of SS and RoT in daily life communication was not controlled. Furthermore, the degree to which each participant prefers a specific type of SS was not controlled; as it might be possible that one participant prefers verbal SS over non-verbal and vice versa. Similarly, the degree to which each participant prefers a specific timing for receiving SS was also not controlled; as it might be possible that one participant prefers to receive the SS immediately after the end of the utterance than receiving it after a delay of certain seconds. Second, we did not recruit participants with a severe lack of perceived SS and RoT communication issues. Third, all the materials of the experiment were translated into Japanese, with a specific linguistic and cultural background. Therefore, the observed effects do not necessarily guarantee real-world reproducibility. The obtained minimal significant results are limited as we hired the participants having no severe lack of perceived SS and RoT issues in their daily life communication. Therefore, interactive experiments with individuals having a severe lack of perceived SS and RoT issues using the proposed system in a more controlled manner are required to observe the actual potential of the system in practice and draw more decisive conclusions. The observed effect of SS was significant. However, the average values were the same for both clearly distinctive experimental conditions. The possible reason could be that a few of the participants virtually recognized both conditions as dyadic in nature; as apparently, there were only two humans involved in communication alongside a third agent that wasn’t a human but a robot avatar.

Moreover, in switching avatars, since the utterances produced were opinions of operators with a predefined non-verbal social behavior so, there was an absence of additional emotional responses from the third agent. It might be a difficult situation for operators to mentalize the agent. The introduction of a second operator for controlling the third agent could be a possible solution for: 1) decreasing the possibility for operators to virtually recognize both conditions as dyadic in nature and, 2) mentalizing the third agent. In future interactive experiments, consideration of such a factor would also help to gauge the potential of the proposed system. The differences in the personalities of the individuals with respect to the presence or absence of a severe lack of perceived SS and RoT will require defining a proper criterion with the help of a professional e.g., psychiatric or psychologist to categorize the individuals with minor, moderate, and severe lack of perceived SS and RoT. However, such a categorization process will require further preliminary experiments; as there will be a need for modifications in the questionnaires of perceived SS and RoT scales to cover the context of lack of perceived SS and RoT in daily life communication.

Another major limitation of our study is the adoption of the non-verbal behaviors of avatar robots to influence the perceived SS of operators. We did not explore the effects of using verbal or a combination of verbal and nonverbal behaviors of avatar robots on the perceived SS of operators. Moreover, we did not explore the effect of the time of SS provision to operators. Furthermore, we did not verify whether the effect of RoT will continue to increase as the number of avatar robots increase. For simplicity, we focused solely on the effects on the operator’s side. However, the use of the proposed system in real life would also require acceptance from visitors, as their chances of being affected by the operators’ severe lack of SS and RoT in communication are higher.

A part from limitations, we also interviewed the participants at the end of experiment; where they were required to share their positive and negative experiences with us so that we can further explore the potential of the proposed system. A few positive comments are as follows: 1) The voice recognition and utterance features were nice. 2) Two different anonymous voices for each avatar was a nice feature; as it ensured the protection of the personality behind the second avatar. 3) Although the discussion was difficult, however, I liked it. The system was good for heated discussions; where I could say opinions more openly without compromising friendships and showing actual emotions to others. 4) The feature of being anonymous is useful in real-life applications. 5) In case we are busy, the system has the potential to support us. 6) Such a system would help a lot in reducing the shyness in the conversation. 7) The system seems great. I think it will help a younger sportsman to talk more openly with the captain of a team. Similarly, a few negative comments are as follows: 1) Two robot condition was a little bit difficult because the shifting of gaze of the visitor was random, depending on the uttering robot. 2) The switching of robots was a little bit confusing for me. 3) If we can choose the robot to speak, I think, it will further improve the system. 4) Answering the long sentence made the usage of the system difficult for me. 5) One robot condition was easier than two. 6) While typing or speaking, the thoughts about, w*hich robot is going to speak next*? are confusing. 7) If I know which robot is going to speak next, I will be more relaxed.

In addition to these limitations and comments, some challenges would hinder the integration of the proposed system into daily life. In the beginning, it might be challenging to find appropriate individuals with severe lack of perceived SS and RoT issues in communication, and later train them to use such a system in daily life independently. Furthermore, in subsequent stages, it might also be challenging to endure the cost of deploying the system and later bear the maintenance cost together with multiple unforeseen technical and non-technical issues, for which individuals with a severe lack of perceived SS and RoT will be completely dependent on service providers. Despite of aforementioned challenges, the improvement of the lack of perceived SS and RoT should remain the top priority to increase the quality of daily life communication of individuals. To ensure such a priority, the involvement of professionals e.g., psychologists or psychiatrics would be necessary.

## 6 Conclusion

Here, we demonstrated that a robotic video conference system with two teleoperated robot avatars significantly increased the operator’s feelings of RoT and SS in online conversations. While having remote experiences via both robot avatars simultaneously, the operator could speak through any of the robot avatars. Because talking via two teleoperated robot avatars will eventually become a triadic interaction scenario on the visitor’s side, they will be required to abide by the rules of triadic conversation. Such a situation is advantageous for the operators because the individual RoT of each robot avatar will ultimately become the operator’s RoT. Similarly, the SS provided by the robots to each other will also become SS to the operator in front of the visitor throughout the conversation. Moreover, the operator’s SoBA is also expected to increase, as the visitor has to be more attentive to the operator via robot avatars. To verify such expected effects, a field experiment was conducted with RoT, SS, and SoBA as the measured indices. The obtained experimental results indicate the positive effect of using two avatars on the operator’s RoT and SS, but not on the SoBA. In the future, we will examine the effects of using the proposed system for people with a severe lack of perceived SS and RoT issues in communication.

## Supporting information

S1 File English translation of RoT, SS, and SoBA scales. (DOCX)

S2 File An example of triadic conversation involving double avatar condition. (DOCX)

S3 File First topic of conversation used in experiments. (DOCX)

S4 File Second topic of conversation used in experiments. (DOCX)
