# Supplementary material for: Effect of having and switching multiple avatars on the operator’s right to talk and receive social support
Source: PLoS One. 2023 Oct 16;18(10):e0292803. doi: 10.1371/journal.pone.0292803 (PMC10578597; doi:10.1371/journal.pone.0292803)
Supplement: S1 File — (DOCX) [file pone.0292803.s001.docx]

**Right to talk (RoT) scale:**

How did you feel during the conversation? Please evaluate it using the following:

About your right to talk:

1. I could talk normally.
2. I had a fair order to talk.
3. I could talk sufficiently during the conversation.
4. I could express my opinion.
5. The order of the conversation was assigned to me properly.
6. I could express all my sentences without any interruption.

**Social support (SS) scale:**

How did you feel during the conversation? Please evaluate it using the followings:

About social support:

1. They cared for me properly.
2. The member(s) of the conversation expressed affection for me.
3. The member(s) of the conversation respected me.
4. I was treated properly as a member of the conversation.

**Sense of being attended to (SoBA) scale:**

To what extent were you attended to during the conversation? Please evaluate this using the following:

1. I could convey my opinion to the interlocutor.
2. The interlocutor was interested in my opinions.
3. The interlocutor understood my answers.
4. The interlocutor paid attention to me through my avatar (the robot).
5. I could communicate with the interlocutor naturally.
