# Supplementary material for: Effect of having and switching multiple avatars on the operator’s right to talk and receive social support
Source: PLoS One. 2023 Oct 16;18(10):e0292803. doi: 10.1371/journal.pone.0292803 (PMC10578597; doi:10.1371/journal.pone.0292803)
Supplement: S2 File — (DOCX) [file pone.0292803.s002.docx]

**Example:**

**Triadic communication**

**Topic: Car accident**

*Please speak while pointing to the robot with the palm of your hand in the cyan color areas.

** Double Avatar Condition

Visitor: Hello.

Operator: Hi [*uttered by the first avatar*]

Visitor: Today, I would like you to discuss with me the topic, "If you could save either 'a child' or 'two old people' in a traffic accident, which one should you save?".

Operator: Interesting topic! [*uttered by the second avatar*]

Operator: Okay. [*uttered by the first avatar*]

Operator: I am glad to talk about it! [*uttered by the first avatar*]

Visitor: What is your opinion on this topic?

Operator: I think you should save one child. [*uttered by the second avatar*]

Visitor: I see! That opinion is also true. But I think another opinion is also important. I would rather help two old people instead of one child. If two lives and one life are weighed against each other, aren’t the two of them still more important?

Operator: Apparently, yes! [*uttered by the second avatar*]

Operator: Two lives are weighed more than one. [*uttered by the first avatar*]

Operator: However, the child has a higher probability of having a brighter future compared to two old people. [*uttered by the first avatar*]

Visitor: But is anything better than two lives? I don't think so.

Operator: I agree with you but I am thinking from a different perspective. [*uttered by the second avatar*]

Visitor: I understand that the child is younger and still has a long way to go. But would the same be true if it were to go to court later? Do you agree with me that I might be criticized for not saving more lives?

Operator: Yes, I agree because such a situation is also possible where you will be criticized by many individuals in the court. [*uttered by the first avatar*]

Visitor: Is that so? I think this idea will be supported by many other people, and I think it will give you a lot of peace of mind.

Operator: If I fail to save the child, it will also disturb the peace of my mind. [*uttered by the second avatar*]

Visitor: At this stage, wouldn't you like to change your earlier opinion (you should save one child)?

Operator: Yes, I want to change. [*uttered by the second avatar*]

Visitor: Why did you change your mind?

Operator: I think, I will feel double pain if I fail in saving two lives compared to one life. I will have a lot of mental illness issues and I don’t want those. [*uttered by the first avatar*]

Visitor: So, saving two lives is more important than saving one life, even if it's a child?

Operator: Yes, I think so. [*uttered by the second avatar*]

Visitor: Yes! By the way, if you had a car accident in your own life and were forced to choose between "one child" and "two elderly people", what would you do?

Operator: I will save two lives i.e., two elderly people. [*uttered by the first avatar*]

Visitor: I see. Please think realistically about your choice. Do you think it's possible?

Operator: I think yes! [*uttered by the second avatar*]

Visitor: Why do you think so?

Operator: Because I am an adult and powerful enough to help people physically. [*uttered by the first avatar*]

Visitor: Okay. Thank you for your time.
