# Supplementary material for: Effect of having and switching multiple avatars on the operator’s right to talk and receive social support
Source: PLoS One. 2023 Oct 16;18(10):e0292803. doi: 10.1371/journal.pone.0292803 (PMC10578597; doi:10.1371/journal.pone.0292803)
Supplement: S3 File — (DOCX) [file pone.0292803.s003.docx]

**Topic: Car accident**

*Please speak while pointing to the robot with the palm of your hand in the cyan color areas.

Visitor: Hello.

Operator: ………….

Visitor: Today, I would like you to discuss with me the topic, "If you could save either 'a child' or 'two old people' in a traffic accident, which one should you save?".

Operator: ………….

Visitor: What is your opinion on this topic?

Please select your answer below.

- Operator: I think I should save one child. [Branch 1]
- Operator: I think I should save the two old men. [Branch 2]

[Branch 1]

Operator: I think you should save one child.

Visitor: I see! That opinion is also true. But I think another opinion is also important. I would rather help two old people instead of one child. If two lives and one life are weighed against each other, aren’t the two of them still more important?

Operator: ………….

Visitor: But is anything better than two lives? I don't think so.

Operator: ………….

Visitor: I understand that the child is younger and still has a long way to go. But would the same be true if it were to go to court later? Do you agree with me that I might be criticized for not saving more lives?

Operator: ………….

Visitor: Is that so? I think this idea will be supported by many other people, and I think it will give you a lot of peace of mind.

Operator: ………….

Visitor: At this stage, wouldn't you like to change your earlier opinion (you should save one child)?

Please select your answer below.

- Yes, I will change my opinion. [Branch 1-1]
- No, I will not change my opinion. [Branch 1-2]

[Branch 1-1]

Operator: Yes, I change my mind.

Visitor: Why did you change your mind?

Operator: ………….

Visitor: So, saving two lives is more important than saving one life, even if it's a child?

Operator: ………….

Visitor: Yes! By the way, if you had a car accident in your own life and were forced to choose between "one child" and "two elderly people", what would you do?

Operator: ………….

Visitor: I see. Please think realistically about your choice. Do you think it's possible?

Operator: ………….

Visitor: Why do you think so?

Operator: ………….

Visitor: Okay. Thank you for your time.

[Branch 1-2]

Operator: No, I won't change my mind.

Visitor: Why do you think so?

Operator: ………….

Visitor: Does that mean that a child should be prioritized over two elderly people?

Operator: ………….

Visitor: Yes! By the way, if you had a car accident in your own life and were forced to choose between "one child" and "two elderly people", what would you do?

Operator: ………….

Visitor: I see. Please think realistically about your choice. Do you think it's possible?

Operator: ………….

Visitor: Why do you think so?

Operator: ………….

Visitor: Okay. Thank you for your time.

[Branch 2]

Operator: I think I should save the two old men.

Visitor: I see! That opinion is also true. But I think another opinion is also important. I think saving a child should take precedence over saving two old people. I think that the weight of a child's life is different from that of an elderly person.

Operator: ………….

Visitor: In general, I agree that each life is of equal value, but a child may be the only child of the family. In that case, the child should be an important support for the family in the future, and the social meaning will change considerably.

Operator: ………….

Visitor: It is true that old people’s and children’s lives are irreplaceable for their families. However, for the country and people, children may become more important and great people in the future. Even from such a point of view, the significance of saving children is still great.

Operator: ………….

Visitor: It's a little hard to say, but the child's family may support you for the rest of your life for helping the future child of that family. For older people, this may not be the case.

Operator: ………….

Visitor: At this stage, wouldn't you like to change your opinion (you should save the two old men)?

Please choose your answer below.

- Yes, I will change my mind. [Branch 2-1]
- No, I will not change my opinion. [Branch 2-2]

[Branch 2-1]

Operator: Yes, I change my mind.

Visitor: Why did you change your mind?

Operator: ………….

Visitor: So saving a child is more important than saving two old people?

Operator: ………….

Visitor: By the way, if you had a car accident in your own life and were forced to choose between "one child" and "two elderly people", what would you do now?

Operator: ………….

Visitor: I see. Please think realistically about that choice. Do you think it's possible?

Operator: ………….

Visitor: Why do you think so?

Operator: ………….

Visitor: Okay. Thank you for your time.

[Branch 2-2]

Operator: No, I won't change my mind.

Visitor: Why do you think so?

Operator: ………….

Visitor: So, you mean that two lives should be prioritized over one young life, even if it's the old one?

Operator: ………….

Visitor: By the way, if you had a car accident in your own life and were forced to choose between "one child" and "two elderly people", what would you do?

Operator: ………….

Visitor: I see. Please think realistically about that choice. Do you think it's possible?

Operator: ………….

Visitor: Why do you think so?

Operator: ………….

Visitor: Okay. Thank you for your time.
