# Supplementary material for: Effect of having and switching multiple avatars on the operator’s right to talk and receive social support
Source: PLoS One. 2023 Oct 16;18(10):e0292803. doi: 10.1371/journal.pone.0292803 (PMC10578597; doi:10.1371/journal.pone.0292803)
Supplement: S4 File — (DOCX) [file pone.0292803.s004.docx]

**Topic: love vs money**

*Please speak while pointing to the robot with the palm of your hand in the cyan color areas.

Visitor: Hello.

Operator: …….

Visitor: Today, I would like you to discuss with me the topic, " In order to lead a better life, if people were forced to choose between love and money, which one should they choose?"

Operator: …….

Visitor: What is your opinion on this topic?

Please choose your answer below.

- They should choose love. [Branch 1]
- They should choose money. [Branch 2]

[Branch 1]

Operator: they should choose love.

Visitor: I see! That opinion is also true. But I think another opinion is also important. I think they should choose money over love to live a better life. I think there are so many things that can only be obtained with money, don't you think so?

Operator: …….

Visitor: Still, considering the future life, I think it would be more beneficial to choose money, isn't it?

Operator: …….

Visitor: In fact, I think money can buy almost everything. In life, for example, you can buy a house, a car, and other luxuries with money. Would you agree?

Operator: …….

Visitor: I think love will come later. This is because if you have money, you will be financially stable, and you will be freed from the worry that you may not have enough money in your life. Then, I think, you will consider love seriously and assign time for it. Isn't that, right?

Operator: …….

Visitor: At this stage, wouldn't you like to change your previous opinion (choosing love over money)?

Please choose your answer below.

- Yes, I will change my opinion. [Branch 1-1]
- No, I will not change my opinion. [Branch 1-2]

[Branch 1-1]

Operator: Yes, I change my mind.

Visitor: Why did you change your mind?

Operator: …….

Visitor: So you're saying you should choose money over love?

Operator: …….

Visitor: By the way, if in your real life, you had to choose between love and money, what would you do?

Operator: …….

Visitor: I see. Please think realistically about the choice. Do you think it's possible?

Operator: …….

Visitor: Why do you think so?

Operator: …….

Visitor: Okay. Thank you for your time.

[Branch 1-2]

Operator: No, I won't change my mind.

Visitor: Why do you think so?

Operator: …….

Visitor: So, you're saying that choosing love is more important than choosing money?

Operator: …….

Visitor: By the way, if in your real life, you had to choose between love and money, what would you do?

Operator: …….

Visitor: I see. Please think realistically about the choice. Do you think it's possible?

Operator: …….

Visitor: Why do you think so?

Operator: …….

Visitor: Okay. Thank you for your time.

[Branch 2]

Operator: they should choose money.

Visitor: I see! That opinion is also true. But I think another opinion is also important. I think it's important to choose love over money to live a better life. I think there are so many things that can only be obtained through love, don't you think so?

Operator: …….

Visitor: That's true, but I think that if you live a normal life, you should be able to make a certain amount of money. So, even if you prioritize love, you should be able to lead a mentally healthy life, and I think that will eventually lead to a stable income, don't you think so?

Operator: …….

Visitor: Still, I think, I would be happier if I had a partner who would support me in the difficult process that would be required to prepare such money. Don't you agree?

Operator: …….

Visitor: However, having a partner from an early age allows us to experience various difficulties, pleasures, a sense of accomplishment, and social aspects of life that we would not be able to experience alone. If we become parents in the future, I think, we can be an influential and firm protector, isn't it?

Operator: …….

Visitor: At this stage, wouldn't you like to change your previous opinion (choosing money over love)?

Please choose your answer below.

- Yes, I will change my mind. [Branch 2-1]
- No, I will not change my opinion. [Branch 2-2]

[Branch 2-1]

Operator: Yes, I change my mind.

Visitor: Why did you change your mind?

Operator: …….

Visitor: So, you're saying that love is more important than money?

Operator: …….

Visitor: By the way, if in your real life, you had to choose between love and money, what would you do?

Operator: …….

Visitor: I see. Please think realistically about the choice. Do you think it's possible?

Operator: …….

Visitor: Why do you think so?

Operator: …….

Visitor: Okay. Thank you for your time.

[Branch 2-2]

Operator: No, I won't change my mind.

Visitor: Why do you think so?

Operator: …….

Visitor: So, money is more important than love?

Operator: …….

Visitor: By the way, if in your real life, you had to choose between love and money, what would you do?

Operator: …….

Visitor: I see. Please think realistically about the choice. Do you think it's possible?

Operator: …….

Visitor: Why do you think so?

Operator: …….

Visitor: Okay. Thank you for your time.
